# 2002–2022 Quinolone Resistance in *Escherichia coli* of Swine in Mainland China: A Meta-Analysis

**DOI:** 10.3390/vetsci12040345

**Published:** 2025-04-09

**Authors:** Xuelin Long, Shujun Liu, Runmin Kang, Yue Sun, Mingyue Tian, Lijun Zhao, Changwei Lei, Hongning Wang, Xin Yang

**Affiliations:** 1 Key Laboratory of Bio-Resources and Eco-Environment, Ministry of Education, Animal Disease Prevention and Food Safety Key Laboratory of Sichuan Province, College of Life Sciences, Sichuan University, Chengdu 610065, China; xlinlong7872@163.com (X.L.); shujunlk@163.com (S.L.); ayueaxx@163.com (Y.S.); tianmingyue678@126.com (M.T.); zlj17743293660@163.com (L.Z.); leichangwei@scu.edu.cn (C.L.); whongning@163.com (H.W.); 2 Sichuan Animal Science Academy, Chengdu 610066, China; krm@sasa.cn

**Keywords:** *Escherichia coli*, quinolone, meta-analysis, antibiotic resistance

## Abstract

Swine have emerged as significant reservoirs of antibiotic-resistant strains and genes. To establish a sound rationale for applying quinolone antibiotics in the swine industry, a meta-analysis was performed on the resistance rate of swine-derived *Escherichia coli* to quinolone antibiotics in China from 2002 to 2022. We systematically searched through three databases, PubMed, Web of Science, and CNKI, and included 53 eligible studies within the specified timeframe. Resistance rates were calculated using a random-effects model and the Freeman–Tukey double-arcsine transformation. The resistance rates observed were as follows: 37% for levofloxacin (95% CI: 27~47%), 43% for ofloxacin (95% CI: 34~51%), 54% for enrofloxacin (95% CI: 46~62%), and 50% for ciprofloxacin (95% CI: 42~58%). Further subgroup analyses illuminated that the resistance rates were higher in mainland China’s eastern regions than in western regions. Additionally, there was no significant decrease in the resistance rate over time in different periods. From 2002 to 2022, *Escherichia coli* strains from swine in various regions of mainland China exhibited differing degrees of generalized resistance to quinolones. This poses a potential public health risk and underscores the fact that the control of quinolone antibiotics remains a prolonged and ongoing challenge.

## Simple Summary

In this study, we assessed the evolution of resistance to quinolone antibiotics in swine-derived *Escherichia coli* in mainland China from 2002 to 2022 by meta-analysis. Fifty-three studies were included after systematic searches of PubMed, Web of Science, and CNKI databases, and the resistance rates were calculated using random-effects models and Freeman–Tukey double-orthogonal string transformation. The results showed that the overall resistance rates of the four quinolone antibiotics were, in order, enrofloxacin (54%), ciprofloxacin (50%), ofloxacin (43%) and levofloxacin (37%). Subgroup analyses showed that the resistance rate was significantly higher in the eastern region than in the western region, but there was no significant change in the resistance rate before and after 2018, and the testing method did not significantly affect the results. Although China has restricted the use of some quinolone veterinary drugs since 2016, the resistance rate did not decrease significantly, which may be related to the continued use of alternative drugs and the diverse spread of resistance genes. This study reveals the critical situation of quinolone resistance in swine-derived *E. coli*, highlights the public health risk of the cross-species transmission of resistance genes, and provides an important basis for optimizing veterinary antibiotic management.

## 1. Introduction

*Escherichia coli* (*E. coli*) is a conditionally pathogenic bacterium of intestinal commensalism [1]. It helps to generate an effective immune response and maintain metabolic levels [2], but pathogenic *E. coli* often causes postweaning diarrhea and edema in piglets, and colibacillosis can cause high morbidity and mortality in the swine industry [3,4]. Antibiotics are effective treatments for reducing the incidence of this disease, and they are also good growth promoters in animal husbandry [5]. The use of antibiotics has fueled the rapid growth of the farming industry. However, the irrational use of antimicrobial drugs in feed, such as long-term low-dose addition, has led to the development of antimicrobial resistance (AMR) in bacteria of animal origin, which is a serious threat to public health and the sustainable development of the cultivation industry. Multidrug resistance (MDR) is becoming highly prevalent in *E. coli*, and the problem of its drug resistance has become an important issue affecting global public health security. Swine has emerged as a significant and widespread reservoir of antibiotic-resistant strains and genes [6,7].

Quinolone antimicrobials are important broad-spectrum antimicrobial agents against Gram-negative aerobic bacteria that are extensively utilized in clinical practice, and have evolved to the fourth generation since the discovery of the first quinolone drug, nalidixic acid. Quinolones primarily inhibit bacterial DNA gyrase, which hinders DNA replication [8]. Mechanisms of resistance include mutations in the quinolone resistance determining region (QRDR) and plasmid mediated quinolone resistance (PMQR), and all known mechanisms of quinolone resistance can be found in *E. coli* [9]. Studies have demonstrated that quinolones are effective in treating diarrhea caused by *Enterotoxigenic Escherichia coli* (ETEC) [10]. However, with the extensive use of quinolone antibiotics, the resistance of *E. coli* to these antibiotics has become increasingly prevalent [11]. Some studies have shown that *E. coli* strains originating from the livestock industry in China are more resistant to quinolone antibiotics than those in other countries [12]. Quinolone-resistant *E. coli* present in swine feces can spread to the outside environment. Furthermore, these antibiotic-resistant strains of *E. coli* can persist in the external environment for extended periods [13,14,15,16]. This resistance has the potential to spread to humans through the food chain, posing a significant risk to the public environment and human health.

With the rapid development of animal husbandry in China, the hog farming industry has formed a national layout. From the southwestern mountains to the northeastern plains, and from the eastern coast to the northwestern inland, the center of gravity of pig farming layout has been gradually moving northward. However, due to differences in geography, climatic conditions, farming scale methods, and antibiotic use habits, the drug resistance of swine-derived *E. coli* will show regional heterogeneity along with the influences on swine farming production [17,18,19,20].

In this study, we conducted a meta-analysis of nearly two decades of research to investigate the spatial and temporal distribution of quinolone resistance in *E. coli* strains from swine in mainland China.

## 2. Methodology

### 2.1. Search Strategy

This study followed the PRISMA guidelines (Preferred Reporting Items for Systematic Reviews and Meta-Analyses) [21]. The PRISMA checklist was used to ensure that all relevant information was included in the analysis (see Supplementary Data S1). Relevant studies on the resistance of swine-derived *E. coli* to quinolone antibiotics in mainland China were identified. We used medical subject headings (MeSHs) and synonyms from PubMed Central and then created search formulas from three databases: PubMed (https://pubmed.ncbi.nlm.nih.gov/ (accessed on 4 February 2023)), Web of Science (https://webofscience.clarivate.cn/wos/alldb/basic-search (accessed on 4 February 2023)), and CNKI (https://www.cnki.net/ (accessed on 4 February 2023)). The search used “OR” to combine the synonyms and “AND” to combine the subject words. The subject words and the synonyms are as follows: “Swine” or “Pigs” or “Suidae” or “Warthogs” or “War Pigs” or “Wart Hog” or “Phacochoerus”, “*E. coli*” or “*Escherichia coli*”, “Antimicrobial Resistance” or “Drug resistance” or “Bacterial resistance” and “China”. The search covered the period from January 2002 to December 2022.

### 2.2. Selection Criteria

After removing duplicate and review articles, preliminary screening was conducted based on the title and abstract of each article to further exclude irrelevant studies. The following were the study selection criteria: (1) the specifically identified strains were unequivocally *E. coli* strains, (2) the samples originated from swine, (3) the collection times were clearly defined, and the collection period was 2002–2022, (4) the sampling locations were in mainland China, and (5) the antibiotic sensitivity methods and resistance phenotypes were defined.

### 2.3. Exclusion Criteria

The exclusion criteria were as follows: (1) studies with mixed samples of porcine origin with other animal origins, (2) studies that had a sample size of less than 30 samples, (3) those that did not include antibiotic sensitivity tests for quinolone antibiotics, (4) those that had duplicate data, and (5) those that had preselected *E. coli*. For example, before antibiotic sensitization testing, it was determined that *E. coli* contained specific genes.

### 2.4. Data Extraction

Two personnel independently screened the full texts of the eligible studies and extracted the appropriate information, including the first author, year of publication, sampling year, sampling location, sample source, number of strains, antibiotic sensitivity test method, and number of antibiotic resistance strains. The two authors collected and recorded the data on Microsoft Excel 2019 and discussed any disagreements.

### 2.5. Statistical Analysis

Given the anticipated heterogeneity arising from variations in geographic regions, sampling periods, and antimicrobial susceptibility testing methods across studies, a random-effects model was selected a priori to account for these expected differences in effect sizes [8]. And the Freeman–Tukey double-arcsine transformation [22,23] was used to calculate the resistance rate of *E. coli* to each quinolone antibiotic, with a 95% confidence interval. The formula is as follows: t = arcsin (sqrt (r/(n + 1)) + arcsin (sqrt (r + 1)/(n + 1))), set = sqrt (1/(n + 1)), *p* = sin (t/2)^2^, where t is the detection rate after conversion, n is the total number of samples, r is the number of positive samples, set is the standard error, and *p* is the final detection rate. The Freeman–Tukey double-arcsine transformation may stabilize variances better in general, but an additional inverse transformation step is required to restore the original scale, potentially increasing computational complexity [24]. Based on the information extracted from the studies, the I^2^ test was used to assess the heterogeneity of the included studies. I^2^ denotes the proportion of variability in the meta-analysis that is explained by intertrial heterogeneity [25,26]. I^2^ values of approximately 0%, 25%, 50%, and 75% corresponded to no, low, moderate, and high heterogeneity, respectively [27]. If (I^2^ < 50%), there is low or no statistical heterogeneity among the effect sizes of the studies, and a fixed-effects model should be used for analysis. If (I^2^ ≥ 50%), there is significant statistical heterogeneity among the study effects, and a random-effects model should be used for analysis [28]. To assess the publication bias within the included studies, we constructed funnel plots and conducted Egger regression tests. When the *p* > 0.05, we concluded that the results demonstrated no significant publication bias.

All meta-analyses in this study were performed with Stata 17.0 software.

## 3. Results

### 3.1. Study Screening

**Figure 1:**
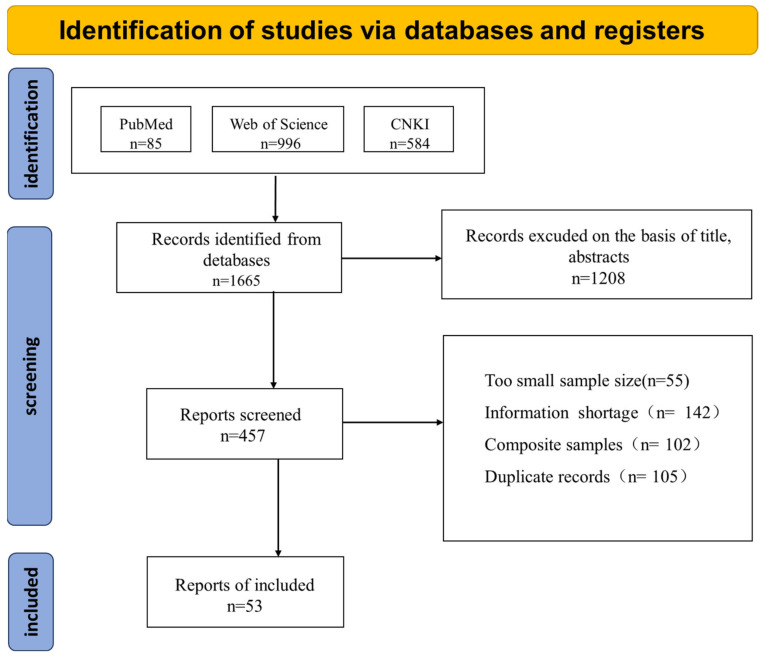
PRISMA flow diagram.

Across three databases, a total of 1665 studies were retrieved and meticulously screened. Subsequently, 1208 studies were excluded based on an initial review of their titles and abstracts. An additional 55 studies were excluded due to insufficiently small sample sizes, 142 due to a lack of necessary information, 102 due to the inclusion of mixed samples, and 105 due to the presence of duplicate records. Ultimately, a total of 53 studies were deemed eligible for inclusion in our analysis. The PRISMA flowchart [29,30] is depicted in Figure 1

In this study, we analyzed a comprehensive dataset consisting of 7916 *E. coli* strains. Based on their highest abundance within the dataset, we selected four quinolone antibiotics for investigation: levofloxacin, ofloxacin, enrofloxacin, and ciprofloxacin. The dataset included strains originating from seven major geographic regions across China, namely the North, East, South, Central, Northeast, Northwest, and Southwest. Information about the selected studies is summarized in Supplementary Data S2.

### 3.2. Quinolone Resistance in Swine Escherichia coli

**Figure 2:**
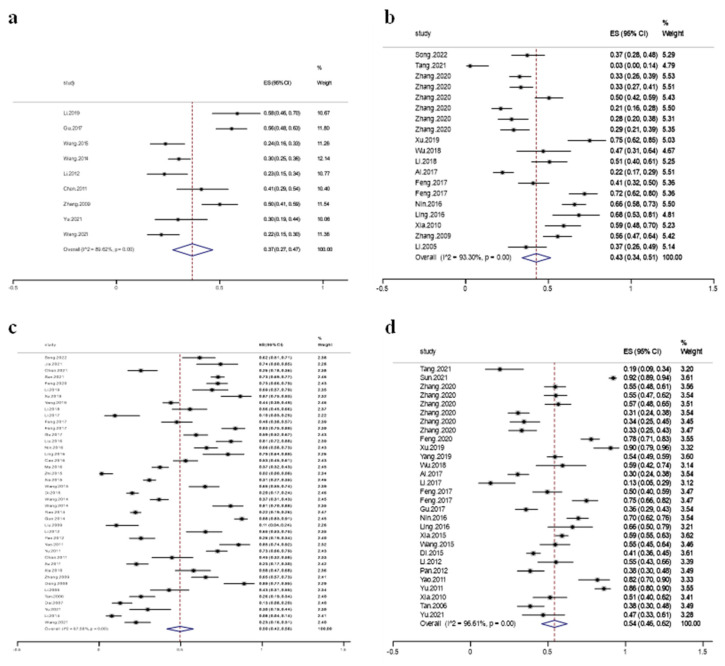
Meta-analysis of the resistance rate of *E. coli* to quinolone antibiotics. (**a**) Levofloxacin; (**b**) ofloxacin; (**c**) ciprofloxacin; (**d**) enrofloxacin.

The data from the included studies were combined and analyzed, and a random effects model was used. The results showed that *E. coli* was universally resistant to the four quinolone antibiotics, with an overall resistance rate ranging from 37% to 54%. The resistance rate of *E. coli* to levofloxacin was 37% (95% CI: 27~47%), that to ofloxacin was 43% (95% CI: 34~51%), that to enrofloxacin was 54% (95% CI: 46~62%), and that to ciprofloxacin was 50% (95% CI: 42~58%) (Figure 2). Notably, *E. coli* demonstrated the highest resistance rate against enrofloxacin, followed by ciprofloxacin and ofloxacin, and the lowest against levofloxacin. In addition, sensitivity analyses showed no significant change in the results after removing the more heavily weighted studies.

ES, resistance rate estimate; CI, confidence interval. The small squares represent the study estimates (the size of the square reflects the study’s statistical weight, and the horizontal lines represent 95% CIs). The hollow diamonds represent summary estimates with corresponding 95% CIs.

### 3.3. Publication Bias

**Figure 3:**
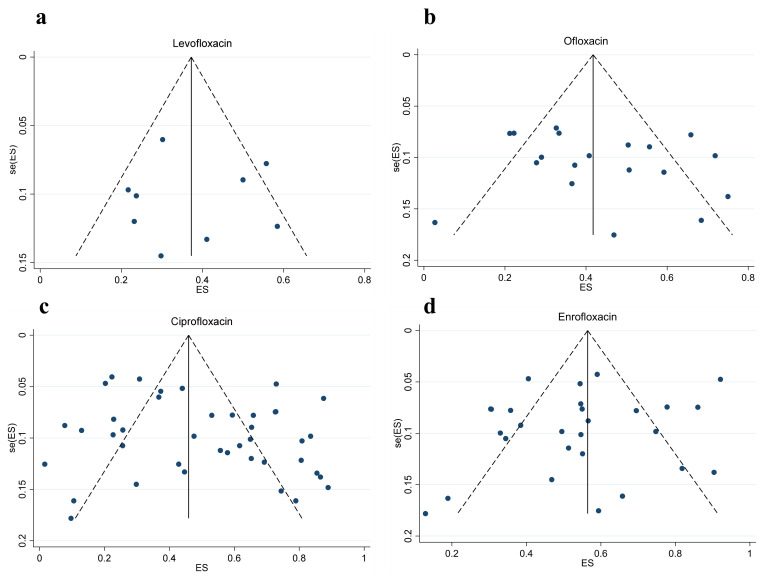
Funnel diagram of quinolone antibiotics. (**a**) Levofloxacin; (**b**) ofloxacin; (**c**) ciprofloxacin; (**d**) enrofloxacin. Funnel plot for assessing potential publication bias. The x-axis represents the standardized mean difference, and the y-axis represents the standard error of the effect size. The solid line indicates the combined effect size, while the dotted line represents the 95% confidence interval of the expected symmetric distribution. The scattered points represent each included study. The symmetric distribution suggests no significant publication bias.

The funnel plot results showed no significant publication bias for levofloxacin, ofloxacin, enrofloxacin, and ciprofloxacin. Moreover, Egger tests for the included studies to detect publication bias (levofloxacin (*p* = 0.997), ofloxacin (*p* = 0.365), enrofloxacin (*p* = 0.294) or ciprofloxacin (*p* = 0.115)) also confirmed this result [31]. Therefore, the results were reliable (Figure 3).

### 3.4. Subgroup Analysis

Subgroup analyses were performed to explore the sources of heterogeneity [32]. Due to the significant volume of data pertaining to ciprofloxacin and enrofloxacin, we chose these antibiotics as representatives for conducting a subgroup analysis. The results are presented in Table 1 and Table 2.

These studies were categorized into three subgroups: antibiotic sensitivity test method, time, and different regions of China. The results showed that the resistance rate of *E. coli* to enrofloxacin was low in central and southwestern China and relatively high in southern and northeastern China. Compared with other regions, *E. coli* in northwestern and southwestern China had a lower resistance rate to ciprofloxacin, and a relatively high rate of resistance was found in southern China. The resistance rate of porcine *E. coli* to both enrofloxacin (*p* = 0.89) and ciprofloxacin (*p* = 0.89) exhibited no statistically significant change before and after the year 2018. Neither the disk diffusion nor the dilution method exhibited a significant impact on the sensitivity of ciprofloxacin (*p* = 0.77) or enrofloxacin (*p* = 0.20).

## 4. Discussion

The prevalence of antimicrobial resistance to quinolones is high among clinical strains in China [33]. Quinolones have long been used for the treatment of bacterial diseases caused by *E. coli* due to their powerful antibacterial effects. With increasing concern about food safety, the State has conducted safety assessments of some quinolones and concluded that lomefloxacin, pefloxacin, ofloxacin and norfloxacin may pose potential risks to the cultivation industry and human health. Therefore, China ceased the production of various salts and esters of four raw materials, lomefloxacin, pefloxacin, ofloxacin, and norfloxacin, and various preparations thereof in 2016, which are used in food animals. Furthermore, since 2017, China has prohibited the operation and utilization of various salts, esters, and preparations containing these four raw materials in the context of food animal applications [30]. However, our results revealed no significant decrease in *E. coli* resistance to ciprofloxacin or enrofloxacin. This result is consistent with previous studies [34]. This could be attributed to the continued use of ciprofloxacin and enrofloxacin in veterinary medicine as alternative quinolones following the discontinuation of ofloxacin and norfloxacin. Due to the lack of continuous resistance surveillance data for ofloxacin after its ban in 2017 in the included literature of this study, and the fact that norfloxacin was not included in the statistics due to an insufficient amount of data, we were not able to directly assess the dynamics of the resistance rates of these two drugs. A study analyzing the prevalence of quinolone resistance genes over the decades from the 1990s to the 2010s reports that the prevalence of the *qnr*B and *qep*A genes has remained low, while the prevalence of the aac (6′)-Ib-cr genes has declined slightly. However, the study noted an increase in the prevalence of *oqx*AB and *qnr*S genes [34]. Therefore, given the increasing diversification of quinolone resistance genes and the extended half-life of quinolones [35], the swine industry can only effectively reduce the prevalence of these resistance genes through sustained, long-term treatment regimens that do not use quinolones.

Our study revealed that resistance to quinolone antibiotics was prevalent in swine-derived *E. coli*, and the resistance rates, from highest to lowest, were those of enrofloxacin, ciprofloxacin, ofloxacin, and levofloxacin. In terms of antibiotic resistance mechanisms, there are mainly plasmid-mediated quinolone resistance mechanisms (qnr, aac (6′)-Ib-cr, OqxAB) and the resistance mechanisms caused by *gyr*A and *par*C chromosome mutations [36,37,38]. The main cause of quinolone resistance in *Escherichia coli* is mutations in the genes of the quinolone resistance-determining region, especially mutations at amino acid positions 67–106 encoded by the *gyr*A gene and mutations at amino acid positions 71–110 encoded by the *par*C gene [39,40]. Quinolone antibiotics are incompletely metabolized in swine and may remain in the environment, and these plasmid-mediated determinants promote the horizontal spread of quinolone resistance among community-acquired Gram-negative bacterial species (such as *Neisseria* spp. and *Haemophilus* spp.) and strains from their surrounding environments [41,42,43]. Some studies have also shown that if an isolate is resistant to one quinolone, it will be resistant to other quinolones [44]. Therefore, resistance to quinolones is a threat to public health.

Different antibiotics are used in different regions, which may lead to the presence of geographically heterogeneous resistance. The results of our subgroup analysis showed that the rates of *E. coli* resistance to ciprofloxacin and enrofloxacin varied across regions and were relatively greater in the eastern region than in the western region. This is consistent with previous studies [45]. Studies in mainland China are subject to spatial limitations. The swine farming industry in China is predominantly concentrated in the Sichuan, Henan, and Jiangsu provinces. Consequently, the majority of research efforts have focused on southwestern and central China, where swine populations are dense. And data from northwestern China, where swine populations are relatively sparse, have been infrequently reported [46]. According to statistics, quinolone antibiotics have been among the three most common veterinary antibiotics in China in recent years [47], but there was still no significant decrease in the resistance rate over time during the different periods. This suggests that the overall selective pressure on quinolone antibiotics has not been reduced by the banning of some of these drugs. Therefore, the control of quinolone antibiotics remains a long-term process.

This study is a meta-analysis of uncontrolled dichotomous data without a control group, so its heterogeneity is often large [48]. The high heterogeneity observed (I^2^ > 50%) aligns with our initial expectations, as factors such as regional antibiotic usage patterns and methodological variations were hypothesized to influence resistance rates differentially across studies. Despite the heterogeneity present among our studies, a globally dependable conclusion can still be derived by selecting an appropriate model and data transformation method. The Freeman–Tukey double-arcsine transformation was chosen for its robustness in handling small samples, extreme proportions, and highly heterogeneous data, and was highly compatible with the distributional characteristics of resistance rates of *E. coli* of porcine origin in the study. Despite the computational complexity and difficulty of interpretation, its methodological advantages ensure the reliability of the meta-analytic results. The variations in heterogeneity observed within our studies could potentially stem from the influence of the analytical configurations associated with each study factor. The heterogeneity of this study stems mainly from the geographic, temporal, and methodological diversity of the Chinese pig farming industry. Although these differences were partially corrected by random effects modeling and Freeman–Tukey double-arcsine transformation, data imbalance between the eastern and western regions and inconsistent testing standards may still affect the stability of the results. However, subgroup analyses showed that the main findings (e.g., high resistance rates in the east, non-significant temporal trends) were consistent across subgroups, suggesting that the core findings are robust. The emergence and persistence of resistant bacteria may also be influenced by various factors, including herd size, farming practices, sampling methodologies, sample size, and the spatial boundaries associated with each swine [19,49]. For instance, while resistance surveys typically fail to mention the age of the swine, several studies have indicated that the age of the swine is also a determinant of resistance [50]. Bacteria in piglets appear to have a greater percentage of resistance because *E. coli* can more easily and rapidly colonize the intestines of younger animals, and bacteria in their intestines have greater potential for resistance transfer [51]. However, such studies were not included in the subgroup analyses because there are fewer of these or because they have not been shown in studies. Additionally, we are lacking in continuous research across the longitudinal dimension of time. Although this is difficult to achieve, more detailed information should be indicated in the follow-up antibiotic monitoring process, which is very important for monitoring results analysis.

## 5. Conclusions

In this study, *E. coli* strains of porcine origin exhibited different degrees of generalized resistance to quinolones in various regions of mainland China from 2002 to 2022. The resistance rates to four quinolones (levofloxacin, ofloxacin, enrofloxacin, and ciprofloxacin) were in the range of 37~54%. Due to the limited availability of studies, it was infeasible to meticulously track the spatial and temporal characteristics of the resistance phenotype exhibited by *Escherichia coli* against quinolone antibiotics. Consequently, there is a pressing need to establish a more comprehensive resistance monitoring system to procure more precise and dependable resistance rates.

## Figures and Tables

**Table 1:** Subgroup analysis of the resistance rate of *E. coli* to enrofloxacin.

	Subgroups	No. of Study	Resistance Rate (95% CI)	Heterogeneity Test	
				*p*	I^2^
Area	North China	2	0.55 (0.47, 0.62)	NA	NA
	East China	4	0.54 (0.42, 0.66)	0.00	89.28%
	South China	4	0.68 (0.53, 0.80)	0.00	88.08%
	Central China	13	0.47 (0.34, 0.59)	0.00	95.75%
	Northeast China	1	0.82 (0.70, 0.90)	NA	NA
	Northwest China	3	0.66 (0.33, 0.92)	NA	NA
	Southwestern China	2	0.38 (0.32, 0.45)	NA	NA
method	dilution method	15	0.49 (0.37, 0.61)	0.00	97.66%
	disk diffusion method	14	0.60 (0.48, 0.70)	0.00	94.18%
time	after 2018	26	0.54 (0.48, 0.61)	0.00	94.27%
	before 2018	3	0.50 (0.05, 0.96)	NA	NA

NA: the data are not available because the number of studies did not exceed 3.

**Table 2:** Subgroup analysis of the resistance rate of *E. coli* to ciprofloxacin.

	Subgroups	No. of Study	Resistance Rate (95% CI)	Heterogeneity Test	
				*p*	I^2^
Area	North China	3	0.46 (0.16, 0.78)	NA	NA
	East China	7	0.51 (0.36, 0.66)	0.00	95.53%
	South China	10	0.58 (0.35, 0.79)	0.00	98.57%
	Central China	10	0.51 (0.33, 0.69)	0.00	96.58%
	Northeast China	2	0.45 (0.40, 0.51)	NA	NA
	Northwest China	5	0.34 (0.16, 0.55)	0.00	98.86%
	Southwestern China	5	0.42 (0.20, 0.66)	0.00	97.28%
method	dilution method	30	0.48 (0.31, 0.64)	0.00	98.79%
	disk diffusion method	12	0.50 (0.42, 0.59)	0.00	96.25%
time	after 2018	5	0.51 (0.27, 0.75)	0.00	97.22%
	before 2018	37	0.50 (0.42, 0.58)	0.00	97.57%

NA: the data are not available because the number of studies did not exceed 3.

## Data Availability

Data are contained within the article.

## Supplementary Materials

The following supporting information can be downloaded at: https://www.mdpi.com/article/10.3390/vetsci12040345/s1.
